# A case study of a phytoplankton bloom triggered by a tropical cyclone and cyclonic eddies

**DOI:** 10.1371/journal.pone.0230394

**Published:** 2020-04-10

**Authors:** Haibin Lü, Xingyu Zhao, Jialong Sun, Guozhen Zha, Jingyuan Xi, Shuqun Cai

**Affiliations:** 1 Jiangsu Key Laboratory of Marine Bioresources and Environment / Jiangsu Key Laboratory of Marine Biotechnology, Jiangsu Ocean University, Lianyungang, Jiangsu province, China; 2 State Key Laboratory of Oceanography in the Tropics, South China Sea Institute of Oceanology, Chinese Academy of Sciences, Guangzhou, Guangdong province, China; 3 State Key Laboratory of Satellite Ocean Environment Dynamics, Second Institute of Oceanography, Ministry of Natural Resources, Hangzhou, Zhejiang province, China; 4 Southern Marine Science and Engineering Guangdong Laboratory, Guangzhou, Guangdong province, China; Guangzhou University, CHINA

## Abstract

Strong tropical cyclone (TC) Ockhi occurred in the southeastern Arabian Sea (AS) in 2017. Ockhi greatly changed the oceanic conditions and induced large variation in chlorophyll-*a* (Chl-*a*). The dynamic mechanisms of the long-term phytoplankton bloom after the passage of the TC were investigated in this study. Prominent surface ocean responses, e.g., decreasing temperature and salinity, were identified from Argo data by comparing the pre- and post-conditions of the TC. A phytoplankton bloom was observed in southeastern AS after the passage of TC Ockhi within the area of (11°N-14°N, 67°E-70°E) and lasted for seven days. Interestingly, there were two weaker cyclonic eddies, with an average vorticity of less than 0.14 s^-1^, on the TC trajectory from November 28 to December 2. As Ockhi approached, strong vertical mixing occurred on December 3, increasing the eddy vorticity to 0.26 s^-1^. After the passage of Ockhi, both eddies, with a two-day oscillation period, were substantially enhanced. Especially from December 11 to 16, the vorticity above 70 m was as high as 0.2 s^-1^ in the thermocline. Because of the high photosynthetically available radiation (PAR) and low precipitation, the enhanced cyclonic eddies induced upwelling for the entire thermocline for over ten days and uplifted nitrates into the mixed layer. This study offers new insights on the influence of eddies in regulating the impacts of typhoons on Chl-*a*, and the results can help evaluate typhoon-induced biological responses in the future.

## Introduction

Tropical cyclones (TC) are strong interactions between the atmosphere and the ocean, and warm surface water supplies energy to TCs [1]. During the TC landfall, strong winds, torrential rains, and storm surges always occur and are very severe threats to human life and property.

The northern Indian Ocean is a TC occurrence area comprised of the Arabian Sea (AS) and Bay of Bengal (BoB). Because five to six times as many TCs occur in the BoB than in the AS [2], the TCs in the BoB have received more attention than those in the AS. In the months of October to December and May to June, the high sea surface temperature (SST), thermodynamically unstable atmosphere and weak tropospheric wind shears are favorable for TC development in the BoB [3]. The BoB is a semi-enclosed basin with strong haline stratification in the near-surface layers due to the influx of freshwater from rivers and monsoonal rainfall [4, 5]. The SST is a crucial parameter in the propagation process of TCs. The decrease in the SST triggered by the TC over the BoB has been observed to vary from 0.3 to 3°C according to the strength and path of the TC [6–11]. A significant increase in Chl-a concentrations was also found following a cyclone in the BoB [12–14]. Some studies suggested that the main contribution to the chlorophyll blooms in the BoB was strong Ekman pumping by the TC [13, 15], and some authors believed that the main contribution was ocean currents and eddies associated with a cyclone strong enough to erode stratification [16].

**Fig 1:**
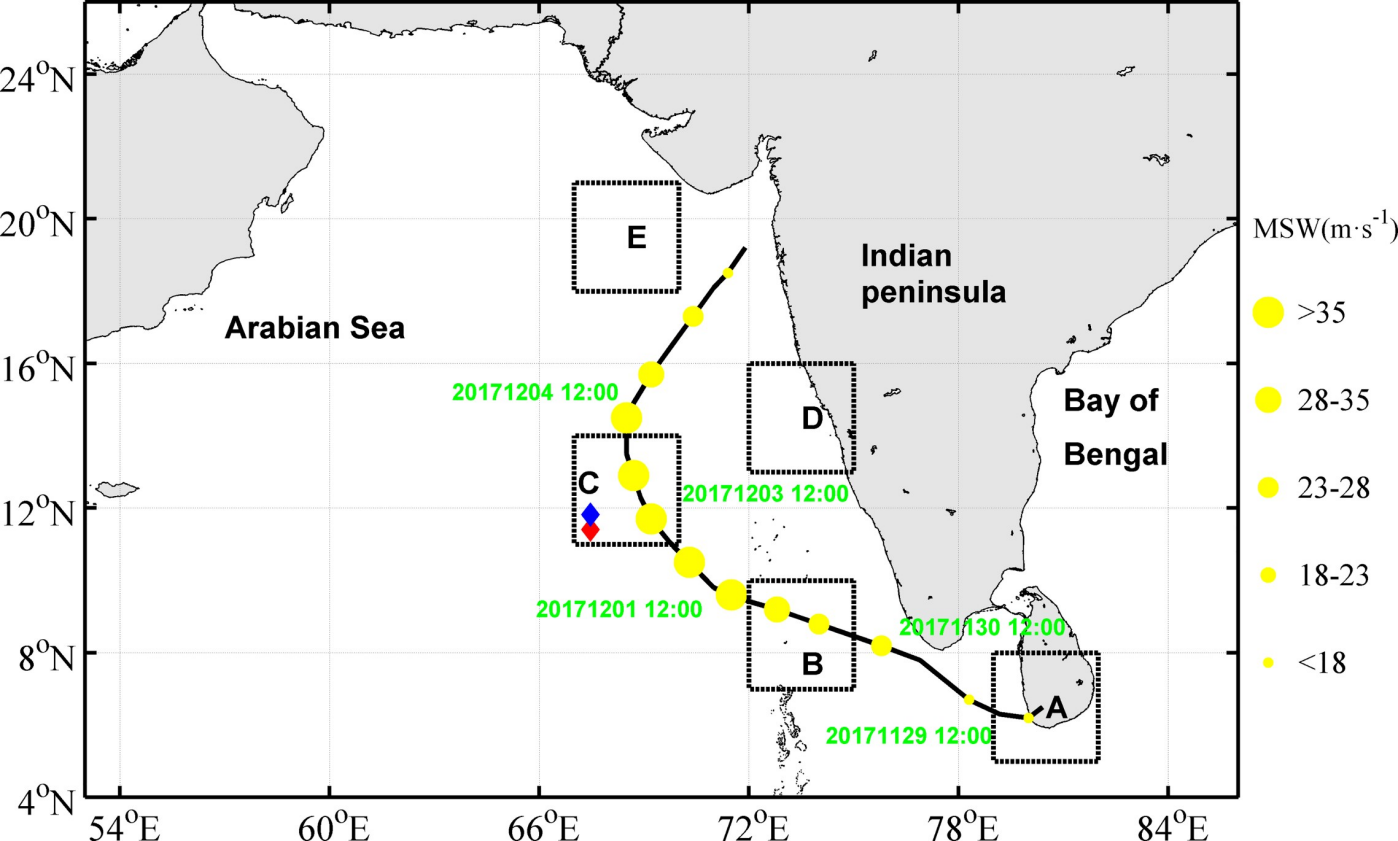
Track of TC Ockhi. Locations of the TC center are indicated by the yellow circles in the time format of year-month-date -hour. Diamonds mark the positions of the Argo floats. The circle size represents the maximum wind speed. The five studied boxes are indicated by dashed black squares, i.e., Box A: 5°N-8°N,79°E-82°E; Box B: 7°N-10°N,72°E-75°E; Box C: 11°N-14°N,67°E-70°E; Box D: 13°N-16°N, 72°E-75°E; Box E: 18°N-21°N,67°E-70°E.

On one hand, the response of the upper ocean to TCs in the AS has been researched by relatively few scientists. Rao et al. [17] investigated the effect of cyclone-ocean interactions primarily to understand the process of subsurface warming based on a 3-dimensional Princeton Ocean Model for the eastern part of the AS. Understanding the upper ocean responses to TC is also important for the accurate prediction of TCs [18, 19]. Hence, the upper ocean response to TCs in the AS is worth investigating. On the other hand, TC Ockhi was a unique and interesting TC that was generated south of Sri Lanka on November 29, 2017, lingered around the tip of the Indian Peninsula and made a landfall along the middle of the west coast of the Indian peninsula on December 5, 2017 (Fig 1).

In this study, the influence of TC Ockhi on the phytoplankton Chl-a was investigated in the upper ocean. The data and methodology used in this study are described in Section 2. The results are presented in Section 3, and discussion analyses are presented in Section 4. Finally, the conclusions are summarized in Section 5.

## Data and methodology

### Data

Daily Chl-a and photosynthetically available radiation (PAR) with a 4 km spatial resolution derived from the GlobColor L3 product were used (available at http://hermes.acri.fr/index.php?class=archive). The GlobColour project started in 2005 as an ESA Data User Element (DUE) project to provide a continuous dataset of merged L3 Ocean Colour products. The GlobColour data set provides a large set of merged Ocean Colour products.

The Cross-Calibrated Multi-Platform (CCMP) gridded surface vector winds were produced using satellite, moored buoy, and model wind data and were considered a Level-3 ocean vector wind analysis product. The CCMP V2.0 processing combined Version-7 RSS radiometer wind speeds, ASCAT scatterometer wind vectors, moored buoy wind data, and ERA-Interim model wind fields using a variational analysis method (VAM) to produce four daily maps of 0.25 degree gridded vector winds [20]. In this study, daily CCMP V2.0 gridded surface vector wind products were used from the dataset available at http://www.remss.com/measurements/ccmp.

The near-real-time daily sea surface height (SSH), sea current velocity, sea water temperature and salinity data with high resolutions of (1/12)°×(1/12)° were derived from the merged and gridded products of the global ocean Copernicus Marine Environmental Monitoring Service (CMEMS), which provides in situ temperature and salinity (TS) profiles, sea surface temperature (SST), and sea level data (available at http://marine.copernicus.eu/). As an essential climate variable, it has been widely used in climate change studies [21–24].

The daily accumulated precipitation product was derived from Tropical Rainfall Measuring Mission (TRMM) between NASA and the Japan Aerospace Exploration Agency, which was generated from the 3-hourly TRMM Multi-Satellite Precipitation Analysis (available at https://daac.gsfc.nasa.gov/).

**Fig 2:**
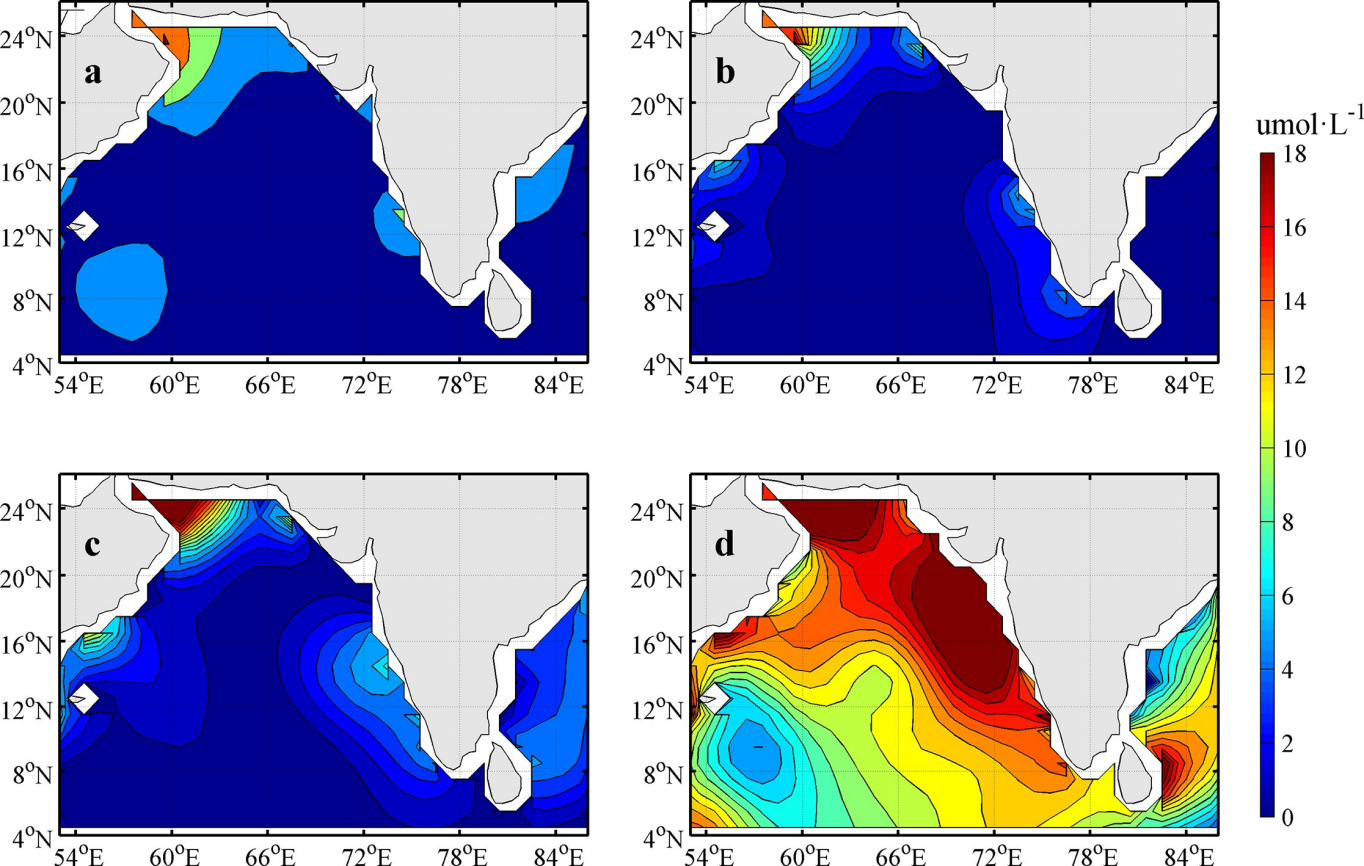
Climatology nitrate concentrations (umol·L^-1^) in December derived from WOA18 (a, b, c and d represent the results at depths of -5 m, -30 m, -40 m and -80 m, respectively).

The 1°×1° resolution climatology nitrate concentration profiles in December were derived from the World Ocean Atlas 2018 (WOA18, https://www.nodc.noaa.gov/OC5/woa18/woa18data.html). WOA18 presents monthly mean data in the 0~ -1500 m layer. The climatology of nitrate concentrations in the study area is shown in Fig 2. The coasts of the map were displayed according to Wessel et al [25].

Temperature and salinity (T/S) profiles for the subsurface stratification were obtained using an Argo float from the Indian Argo project (available at http://www.argo.ucsd.edu). The Argo platform number was 2902128, the cycle numbers were 137 and 138, and their locations were 67.475°E 11.506°N and 67.469°E 11.714°N, respectively (Fig 1). The data were generated on Dec. 4 and Dec. 14, 2017, respectively. The Argo platform locations and data generation time were consistent with the location and timing of the TC passage for analysis.

Track of TC Ockhi was obtained from the Joint Typhoon Warning Center (JTWC) (available at https://www.metoc.navy.mil/jtwc/jtwc.html), which provided typhoon forecasts for the Western Pacific and Indian Ocean basins. Data of TC was at 6-hourly intervals including time, wind speed and central location.

### Methodology

### Ekman pumping velocity (EPV)

Enriquez and Friehe [26] proposed Eqs (1) and (2) to compute the Ekman pumping velocity caused by the spatial variation in wind stress. Since then, the EPV due to vertical mixing triggered by typhoons has been well studied [27, 28]. The velocity of upwelling can be calculated according to Eq (1).

$$w_{E}=\frac{1}{\rho f}(\nabla \times \tau )$$ (1)

where *W*_*E*_ is the Ekman pumping velocity; ρ = 1024kg m^-3^, f and *τ* are the seawater density, Coriolis parameter and wind stress, respectively. The curl of the wind stress can be calculated according to Eq (2).

$$\nabla \times \tau =\frac{1}{R\cos\varphi }[\frac{\partial \tau_{y}}{\partial \lambda }-\frac{\partial }{\partial \varphi }(\tau_{x}\cos\varphi )]$$ (2)

where R, φ and λ are the radius of the earth and the geographic latitude and longitude, respectively; *τ_x_* and *τ_y_* are the zonal and meridional wind stress components, respectively.

### Vorticity

The curl of a sea current vector (u,v) can be calculated based on Eq (3).

$$curl_{z}=\frac{dv}{dx}-\frac{du}{dy}$$ (3)

where *u* and *v* are the two velocity components along x and y directions, respectively.

## Results

### Climatology nitrate distribution in December

Nutrient provides one of the basic conditions for the phytoplankton bloom. The nitrate concentrations at different depths with z = -5 m, -30 m, -40 m and -80 m according to the nitrate climatology data in December are shown in Fig 2. The nitrate distribution in the AS showed three high-concentration zones: the southwest, north and southeast of the AS. Along the west coast of Indian peninsula, high concentrations of nitrates reached 2 μmol·L^-1^ (6 μmol·L^-1^) at a depth of z = -5 m (-40 m). However, the nitrate concentration remained at approximately 18 umol·L^-1^ at a depth of z = -80 m (Fig 2).

### Path of TC Ockhi and wind field

Tropical depressions (10.8–17.1 m·s^-1^), tropical storms (17.2–24.4 m·s^-1^), severe tropical storms (24.5–32.6 m·s^-1^), typhoons (32.7–41 m·s^-1^), severe typhoons (42–51 m·s^-1^) and super-severe typhoons (over 52 m·s^-1^) were classified according to the near-central maximum wind speed (MWS) of TC. TC Ockhi originated at 6.5°N, 81.8°E on November 29, 2017 as a tropical depression, became a tropical storm at 7.5°N, 77.5°E on November 30 and a severe tropical storm at 8.9°N, 73.8°E on Dec. 1. On Dec. 2 06:00 UTC, TC Ockhi strengthened into a typhoon with a maximum wind speed of 42.5 m·s^-1^ and then quickly weakened into a strong tropical storm, a tropical storm and a tropical depression. Ockhi made landfall in the middle of the west coast of the Indian peninsula on Dec. 5 18:00 UTC (Table 1).

**Table 1:** Position, time, intensity and translation speed of TC Ockhi (MSW: Maximum wind speed; TS: Translation speed).

Lat(°N)	Lon(°E)	Time	MSW(m·s^-1^)	TS(m·s^-1^)
5.9	80.8	11/29/06	12.5	
6.2	79.7	11/29/12	12.5	6.2
6.7	78.8	11/29/18	15	4.9
7.2	77.9	11/30/00	17.5	7.6
7.8	76.6	11/30/06	22.5	6.0
8.1	75.8	11/30/12	22.5	5.5
8.5	74.9	11/30/18	22.5	4.8
8.7	74.2	12/01/00	27.5	3.9
8.8	73.3	12/01/06	32.5	4.6
9.2	72.8	12/01/12	32.5	5.3
9.4	72.1	12/01/18	35	3.7
9.3	71.5	12/02/00	37.5	3.5
9.8	71	12/02/06	42.5	2.9
10.5	70.3	12/02/12	42.5	5.0
11.1	69.7	12/02/18	42.5	4.7
11.7	69.2	12/03/00	42.5	4.0
12.3	68.9	12/03/06	37.5	3.4
12.9	68.7	12/03/12	37.5	3.2
13.5	68.5	12/03/18	37.5	3.2
14.5	68.5	12/04/00	35	5.1
14.7	68.7	12/04/06	32.5	2.9
15.5	69.2	12/04/12	30	5.2
16.6	70.0	12/04/18	27.5	5.1
17.7	70.9	12/05/00	22.5	5.1
18.3	71.5	12/05/06	22.5	4.2
18.5	71.4	12/05/12	15	3.6
19.2	71.9	12/05/18	10	4.3

### SSH, sea surface current and SST

**Fig 3:**
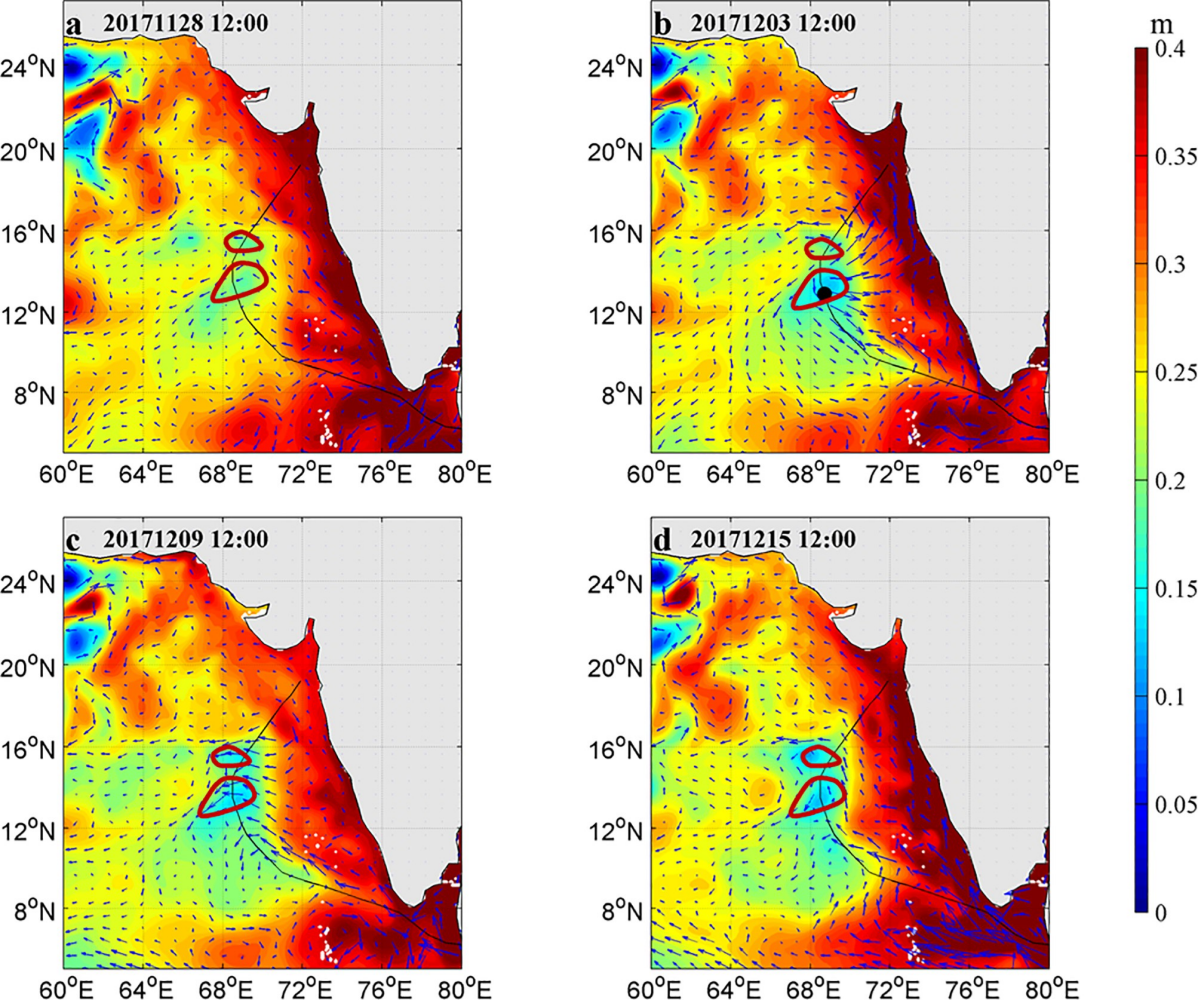
Sea surface current (unit in m·s^-1^) on Nov. 28 (a), Dec. 3 (b), Dec. 9 (c) and Dec. 15 (d). The color bar represents the SSH (unit in m). Track of Ockhi is indicated by the black line. Locations of the TC center are indicated by the black circles. The red rings mark the locations of the two cyclonic eddies.

There were two weaker cyclonic eddies along the longitude 68°E and latitudes ranging from 12°N to 18°N before the arrival of Ockhi (Fig 3A). The positive sea vorticity was calculated to measure the intensity of the cyclonic eddies in the next section (Fig 11). These two cyclonic eddies were enhanced during and after the passage of Ockhi, which maintained for more than ten days (Fig 3D).

**Fig 4:**
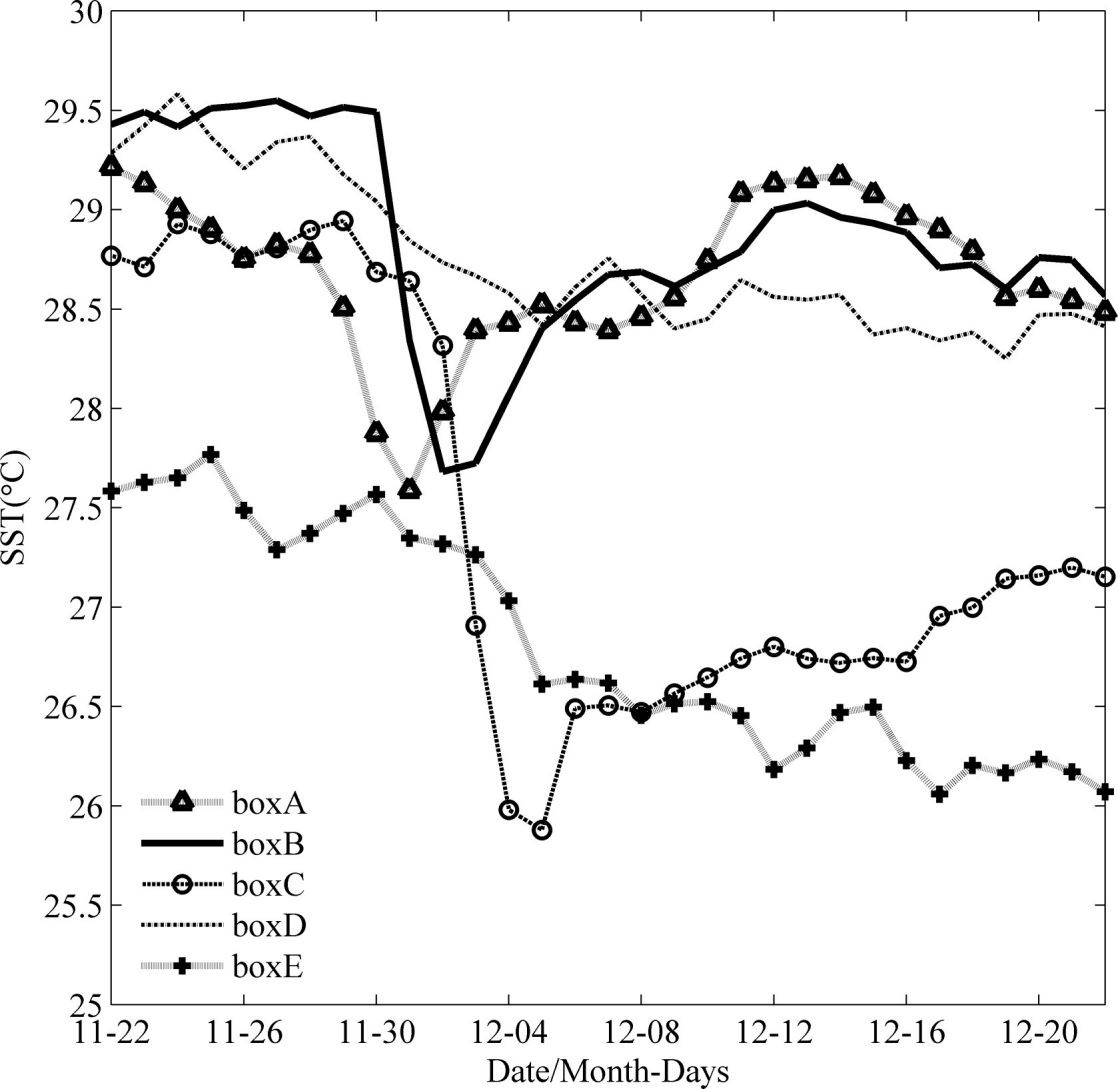
Time series of the spatially averaged SST in boxes A, B, C, D and E from Nov. 22 to Dec. 22.

In response to TC passages, different variables may change differently over time and from location to location, with different memories [29].The minimum SSH along the TC track appeared near the center of the cyclonic eddies. The SSH increased from each eddy’s interior to its periphery, which was consistent with previous studies about the influence of a cyclonic eddy on the SSH [30]. The SST cooling is another significant oceanic response to a typhoon and is influenced by the typhoon strength, speed and other factors [31]. Ockhi had a significant impact on SST near the TC’s path (Fig 4). The SST in box A began to decrease around November 28, with a maximum drop over 1.2°C on December 1. As Ockhi moved to the northwest, the SST in box B (box C) began to decrease around November 30 (December 1), with a maximum drop of approximately 2°C (3°C) on December 2 (December 5). By comparison, the SST in box E decreased more than that in box D. It was interesting that the cooling of SST in box C sustained over 18 days after the passage of Ockhi.

### Chl-a

**Fig 5:**
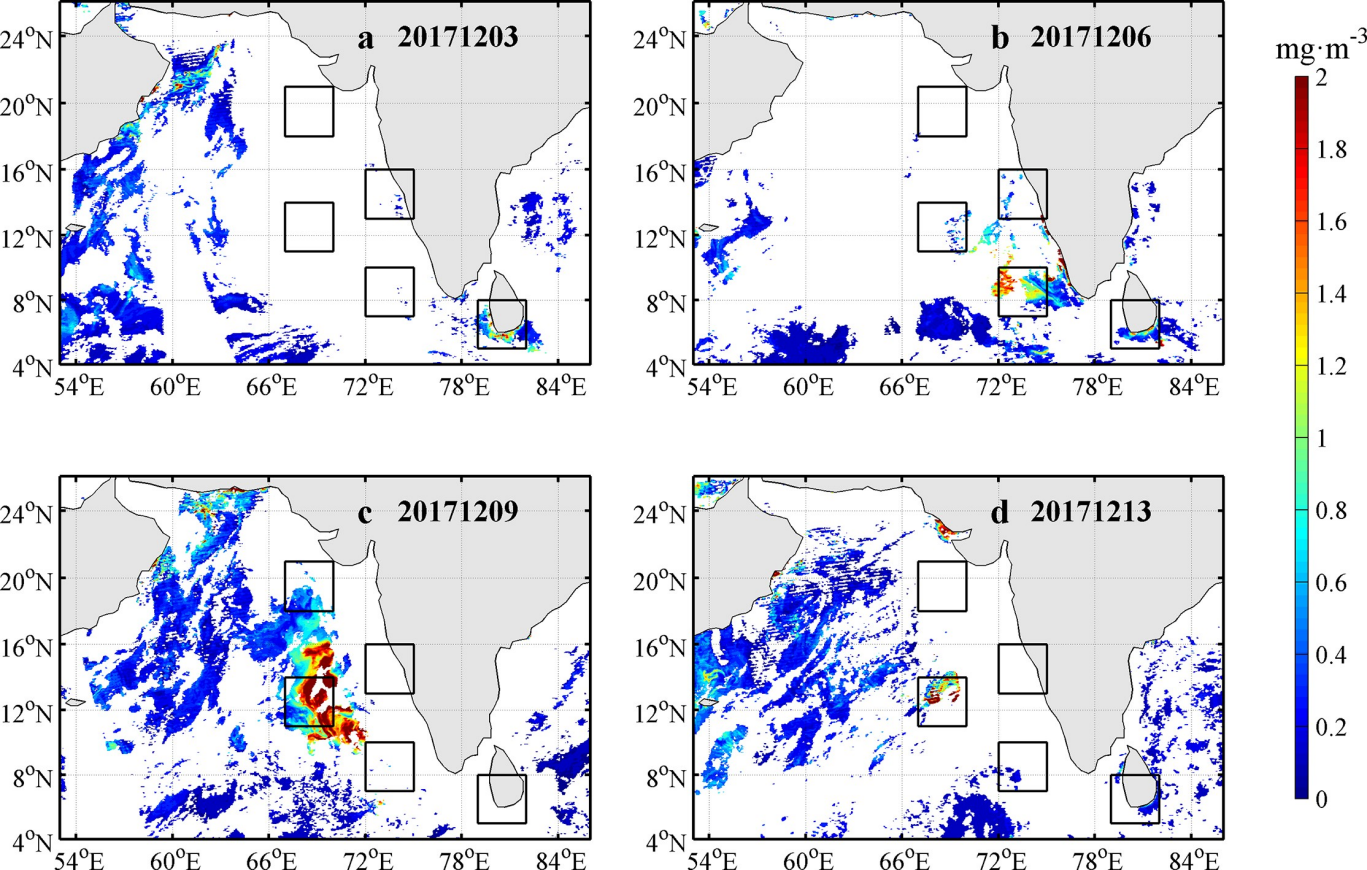
Daily mean Chl-*a* concentration during and after the passage of Ockhi.

**Fig 6:**
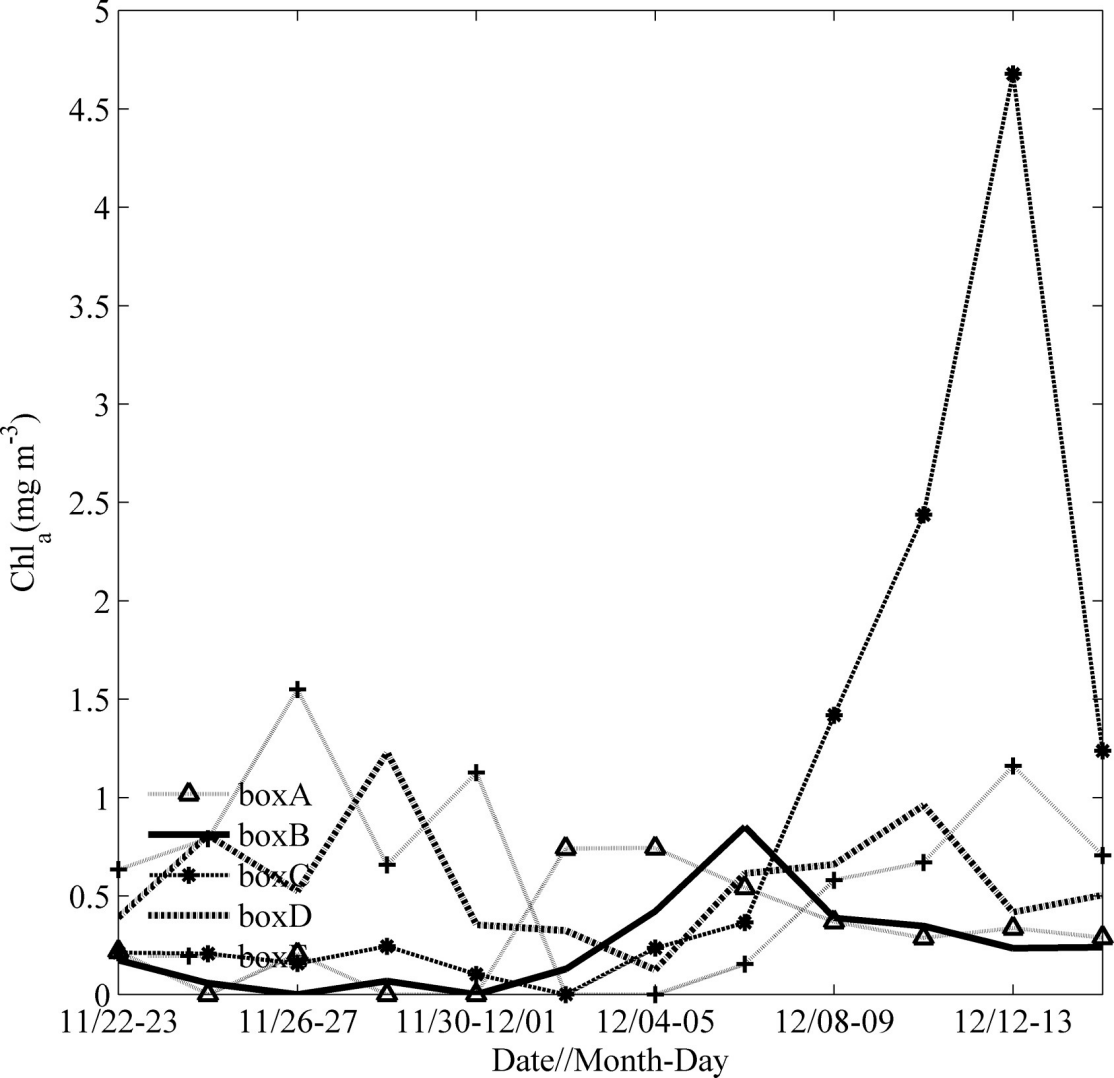
Time series of two-day averaged Chl-a in boxes A, B, C, D and E from Nov. 22 to Dec. 16.

Observations of Chl-a concentration before, during and after the passage of Ockhi are presented in Fig 5. A high Chl-a concentration occurred approximately four days after the passage of the TC center. To avoid the influence of clouds when TC passes, we followed Zhao et al. [27, 31] to calculate the time series of two-day averaged Chl-a to investigate the variation of Chl-a concentration before, during and after the passage of Ockhi in Fig 6. The 2-day-averaged Chl-a concentration time series (Fig 6) are shown for the different boxes. The Chl-a concentration in box A increased obviously around December 2 from 0 to 0.74 mg·m^-3^ and dropped gradually around December 5. After approximately six days, the Chl-a concentration in box B increased gradually around December 6 from 0 to 0.85 mg·m^-3^ and dropped gradually around December 8. The Chl-a concentrations in box C were 0.36, 1.42, 2.44 and 4.68 mg·m^-3^ from December 6^th^ to 12^th^, and dropped obviously around December 14. This phytoplankton bloom continued to increase for 7 days after the passage of Ockhi, as evident by the increase in the sea surface Chl-a concentration by 12 times (Fig 6).

The possible driving mechanisms of the phytoplankton bloom in box C will be analyzed in the next section.

### Precipitation

**Fig 7:**
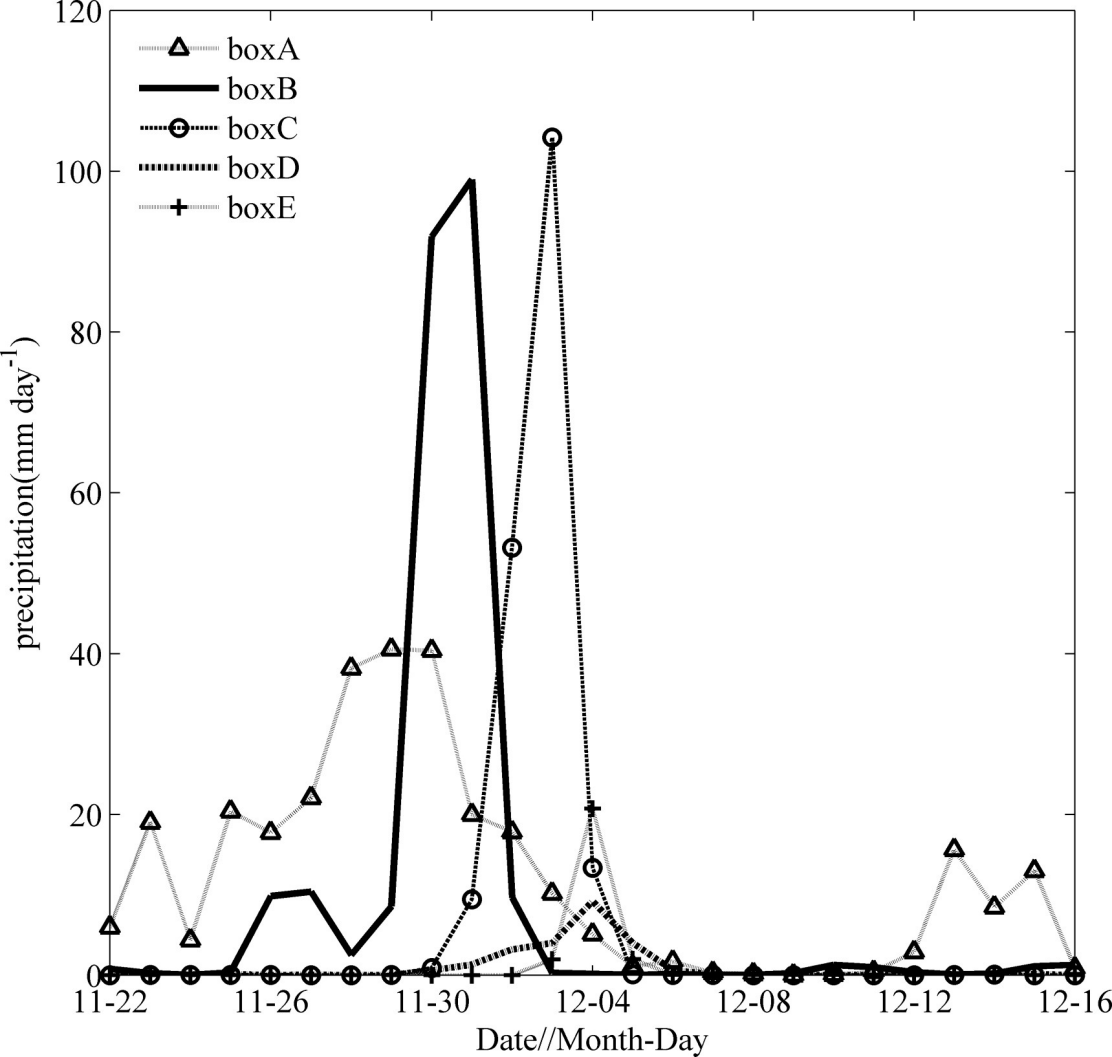
Time series of spatially averaged precipitation in boxes A, B, C, D and E from Nov. 22 to Dec. 16.

The time series of spatially averaged precipitations for different boxes during the TC passage are shown in Fig 7. Ockhi induced significant rainfall near the TC path during its passage. The precipitation in box A began to increase around November 27, with a maximum of 40.54 mm on November 29. As Ockhi moved to the northwest, the precipitation in box B (box C) began to increase around November 29 (December 1), reaching a maximum of 99.01 mm (104.25 mm) on December 1 (December 3). The precipitations in boxes D and E occurred only on December 3 and were no more than 20 mm. It was interesting that the coolings of SST in boxes C and E sustained over 18 days after the passage of Ockhi. On one hand, the heavy rainfall in boxes A, B and C can lead to strong stratification, weaken turbulence [32], and suppress the upward transport of nutrients. On the other hand, there was less precipitation from Dec. 4 to12, that is, rainfall due to the TC was not the most important influence factor for the phytoplankton bloom event in box C.

### Photosynthetically available radiation (PAR)

**Fig 8:**
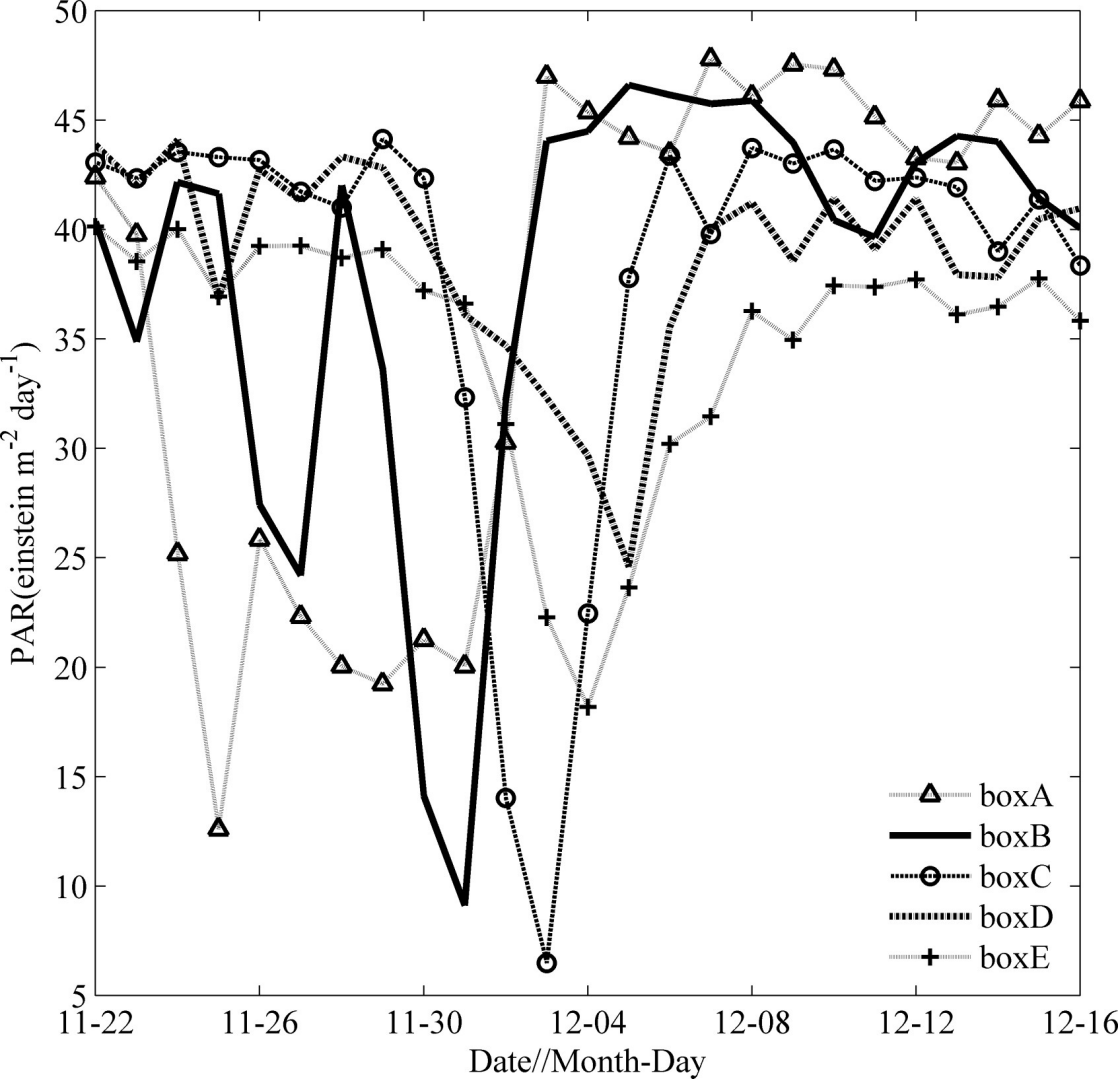
Time series of spatially averaged PAR in boxes A, B, C, D and E from Nov. 22 to Dec. 16.

The time series of spatially averaged PAR for different boxes are shown in Fig 8. Ockhi induced a significant PAR drop near the TC’s path during its passage. The PAR in box A began to decrease around November 25 and remained low until November 30. When Ockhi moved to the northwest, the PAR in box B (box C) reached a minimum on December 1 (December 3). The PARs in different boxes all returned to stable conditions on December 8. PAR began to increase since Dec. 3, with high amount of sunlight entering the euphotic layer; this may be a significant influence factor for the phytoplankton bloom event in box C on December 12.

## Discussion

Generally, with the arrival of typhoon or TC, cyclonic eddies would occur in the upper ocean, which may trigger a phytoplankton bloom. The dynamic mechanism for the bloom of Chl-a has been well studied [27, 31, 41]. Similarly, there also were a cyclonic eddy in box A on Nov.29, box B on Dec. 1 respectively. After the passage of Ockhi, a phytoplankton bloom along the TC track appeared in box A and box B in sequence. We observed that there were high PARs and little precipitation in boxes A-E from Dec. 5–12 (Figs 7 and 8), and the phytoplankton bloom continued to increase in box C for 7 days after the passage of Ockhi, as evident by the increase in the sea surface Chl-a concentration by 12 times (Fig 6). The lag phenomena implied that the Chl-a increase was not induced directly by entrainment mixing from preexisting subsurface chlorophyll. It was unlikely that the increase in Chl-a was caused by horizontal transportation from the surrounding water, where a lower Chl-a patch was observed on Dec. 6. Thus, the phytoplankton bloom was probably due to the phytoplankton growth sustained by new nutrients which were brought into the euphotic zone from below. The possible driving mechanisms for this lagged phytoplankton bloom in box C were analyzed.

### The role of stratification

**Fig 9:**
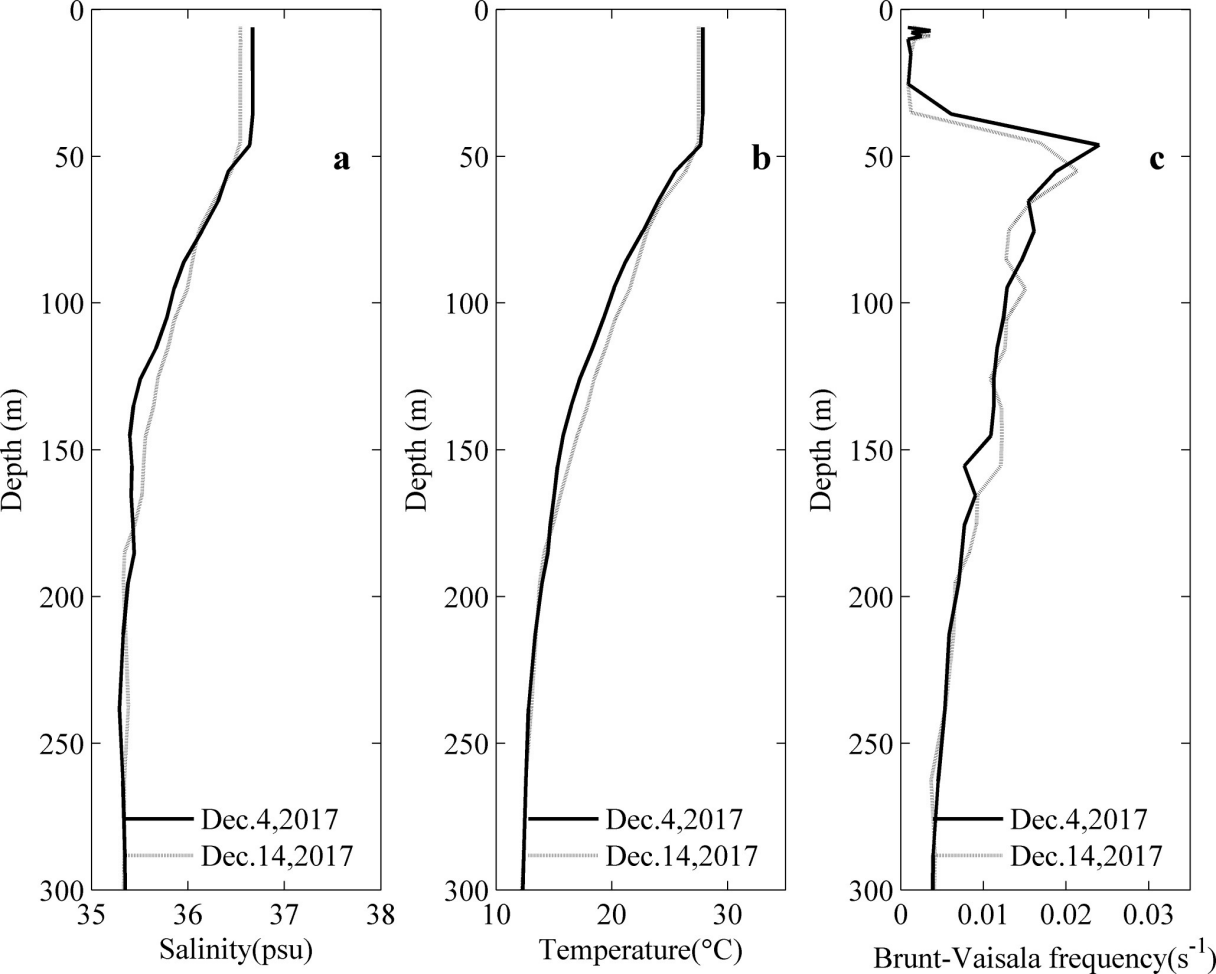
Vertical profiles of salinity (a), temperature (b) and buoyancy frequency (c) from Argo floats before and after the passage of the TC.

The vertical profiles of salinity, temperature and the buoyancy frequency from the Argo floats at two different locations (Fig 1) are shown in Fig 9. The same Argo float remained in box C for more than 10 days, which helped the analysis of the eddy dynamics. The cooling of the upper ocean is an important interaction between TCs and the ocean and mainly depends on the translation speed of the cyclone. For a slow-moving TC, the forcing time in the affected region is relatively longer, thus the local upwelling induced by Ekman pumping can have an overwhelming impact on sea surface cooling [33, 34, 36, 41]. From December 1 to 3, the TC translated with a slow speed from 2.9 m s^-1^ to 4.7 m s^-1^ (Table 1), which might resulted in significant surface ocean cooling [35, 36]. Before and after the passage of Ockhi, the sea water temperature became cooler above the depth of 50 m, decreased from 27.85°C to 27.48°C, and the salinity decreased from 36.67 to 36.54. However, at depths from 50 m to 180 m, the sea water temperature and salinity increased. The thermocline was located at a depth of 46.2 m (55 m) on December 4 (December 14), where the maximum buoyancy frequency was N = 0.024 s^-1^(0.021 s^-1^). Since the mixed layer deepened and stratification in the upper ocean weakened, the accumulation of the phytoplankton in the water volume was promoted, as concluded by Niu et al. [37]. The significant SST cooling in box C had a maximum decrease of approximately 3°C and sustained over 18 days after the passage of Ockhi (Fig 4), which might be caused by the TC and the enhanced cyclonic eddies within the latitude from 12°N to 18°N along the longitude 68°E (Figs 3 and 11). Note that it is the reanalysis data that is used in this study. If in situ SST data were available, the observed SST could be different. This is because most satellite-derived SST products might underestimate the rapid SST drop associated with hurricane/typhoon passage [38]. The SST dropped 4 degrees Celsius within one day after the passage of Hurricane Irma [38].

### Roles of TC and ocean cyclonic eddies

**Fig 10:**
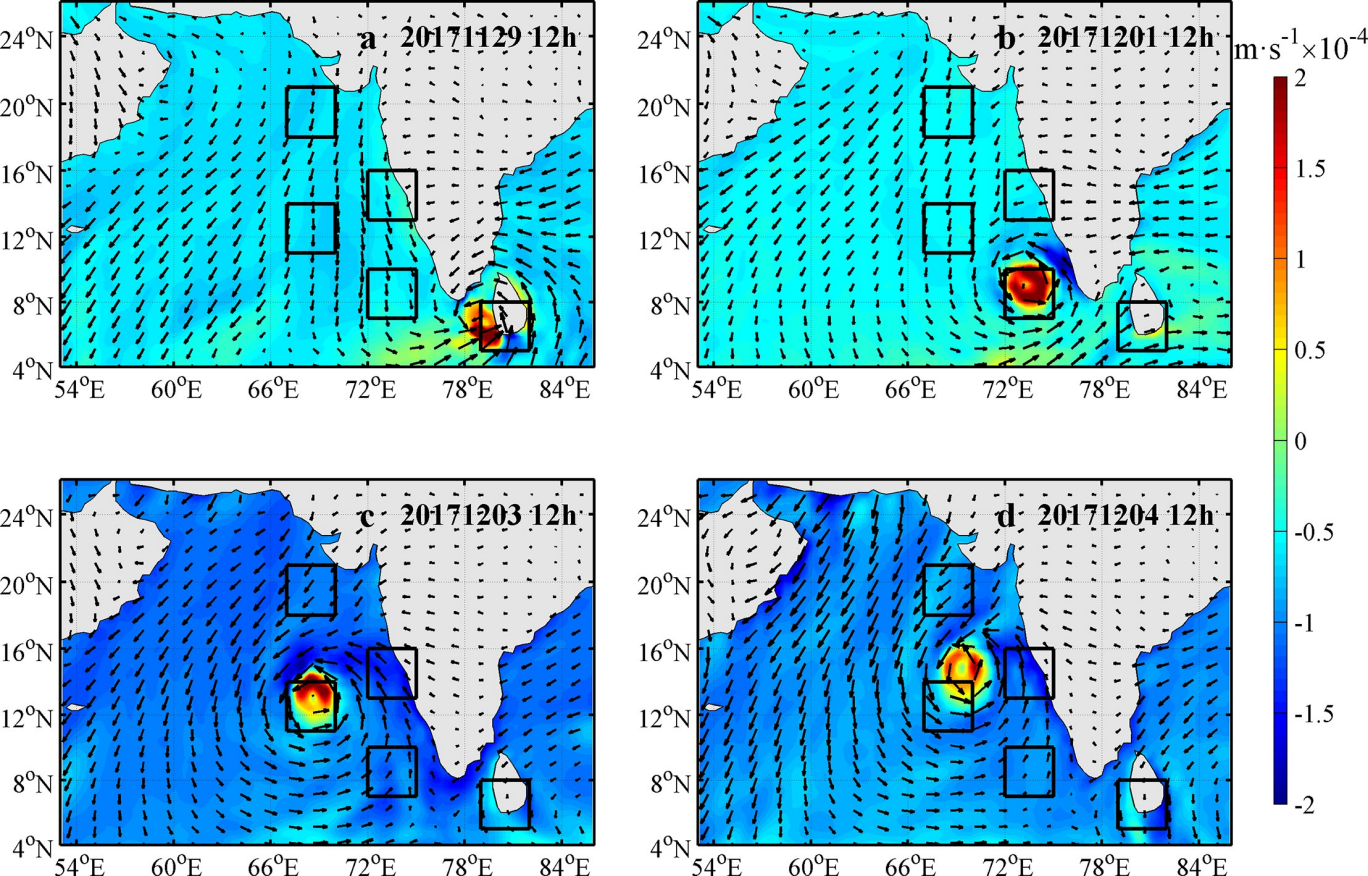
Surface wind speed (arrows) and Ekman pumping velocity (colors) during the passage of the TC.

The mixing and EPV induced by typhoon have been well studied. The Ekman pumping velocity (EPV) can be calculated based on Eqs (1) and (2) [27, 28]. The EPV during the passage period of Ockhi is shown in Fig 10. It took approximately 7 days for the TC to pass over the study area. At 12:00 UTC Nov 29 2017, the upwelling within the vicinity of box A was apparently stronger (>2×10^−4^ m s^-1^), with an eddy diameter greater than 300 km. At the same time, a downwelling ring with a width of approximately 150 km was triggered around the upwelling, which could induce the SSH to lift along the middle of the west coast of the Indian peninsula because of water convergence (Fig 3). The velocity of the upwelling weakened gradually from 12:00 UTC Dec 4 2017. Liu et al. [39] found that a preexisting cold-core eddy might intensify the upper ocean dynamic responses, with a significant increase in nutrients, after the passage of a typhoon. In our study domain, there were two weaker cyclonic eddies within the latitude from 12°N to 18°N along the longitude 68°E before the arrival of Ockhi (Fig 3A). Zheng et al. [40] found that the duration of the sustained influence of the typhoon near its path where the wind speed exceeds 17 m·s^-1^ is greater than the geostropical adjustment time, the mesoscale vortex is more likely to be strengthened where a strong upwelling occurs. Additionally, for typhoons with slow velocity and significant path deflection, the forcing time in the affected region is relatively longer [41]. For Ockhi, its translation speed near box C slowed down (Table 1), and translation direction deflected nearly 90° (Fig 1), which prolonged the forcing time of Ockhi on the cyclonic eddies (Fig 3A). During and after the TC, the cyclonic eddies were significantly enhanced (Fig 3B and 3C). To investigate the effect of the TC and understand the dynamic mechanisms of the phytoplankton bloom, the vorticity was calculated by Eq (3) to measure the intensity of the cyclonic eddies.

**Fig 11:**
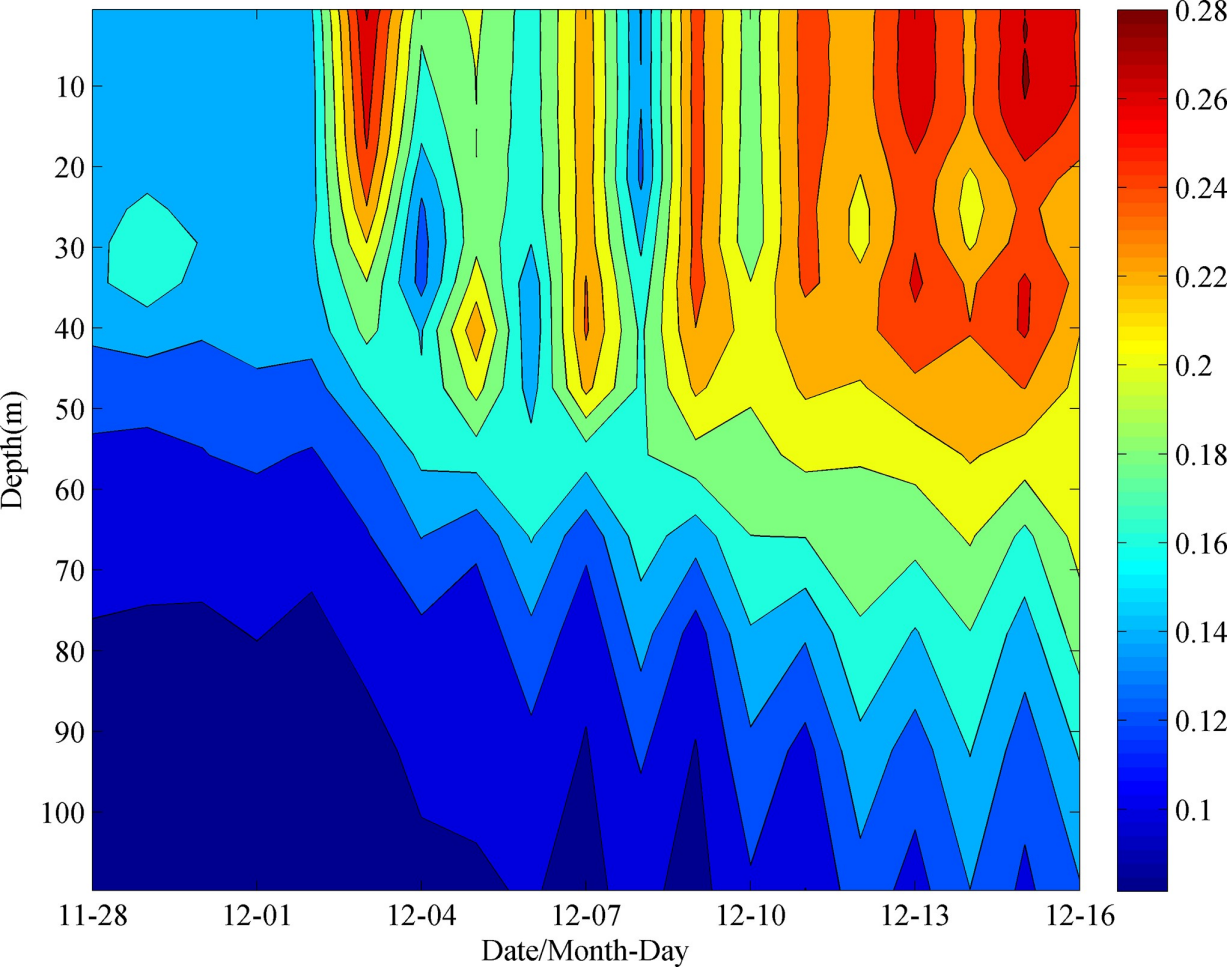
Time series of spatially averaged vorticity in box C from Nov. 28 to Dec. 16.

A time series of the spatially averaged positive vorticity with depth in box C from Nov. 28 to Dec. 16 is shown in Fig 11. As the TC passed on December 3, internal gravity waves with an inertial period of approximately 2 days were generated in the thermocline below the mixed layer (at a depth of 50 m), with large inertial currents and large-amplitude temperature oscillations in the upper ocean. This phenomenon was also observed within the vicinity area (69.2 E, 15.5 N) using a thermistor chain for the TC in June 1998 [17]. Due to the similarities of TCs and the environment in the same region, the oceanic response processes would be similar. At the subsurface, inertial oscillations may take approximately two weeks to reach a normal state because the restoring force is small for internal gravity waves [17]. Before the arrival of Ockhi, there were two weaker cyclonic eddies in box C on November 28 (Fig 3A), with a vorticity of less than 0.14 s^-1^. During the passage of Ockhi, strong vertical mixing occurred on December 3, with a maximum vorticity of approximately 0.26 s^-1^. After the passage of Ockhi, the intensities of the two eddies, which had a two-day oscillation period, were enhanced. Especially from December 11 to 16, the vorticity in box C above a 70 m depth reached 0.2 s^-1^ in the thermocline below the mixed layer, with a maximum value of over 0.28 s^-1^ above the depth of 20 m. Therefore, it is suggested that the two strengthened cyclonic eddies after the passage of Ockhi transported deeper water with rich nutrients into the surface layer through mixing and upwelling, triggering the surface phytoplankton bloom with high PAR and little precipitation (Figs 7 and 8).

## Conclusions

The variation in Chl-*a* and the oceanic conditions in the southeastern AS were analyzed during the passage of Ockhi in 2017. Argo data were used to investigate subsurface ocean responses by comparing the pre- and post-conditions of TC. According to the above analyses and discussion, the following conclusions can be drawn:

1. The sea surface low temperature region near box C (11°N-14°N, 67°E-70°E) lasted over 18 days with a temperature difference of over 3°C after the TC passage. There was a significant increase in the Chl-a concentration for 7 days, with a low value (approximately 0.26 mg·m^-3^) before the TC passage and a 12-fold increase after the TC passage.
2. Before and after the passage of Ockhi, the sea water temperature became cooler above the depth of 50 m, decreased from 27.85°C to 27.48°C, and the salinity decreased from 36.67 to 36.54. However, at depths from 50 m to 180 m, the sea water temperature and salinity increased. The thermocline was located at a depth of 46.2 m (55 m) on December 4 (December 14), where the maximum buoyancy frequency was N = 0.024 s^-1^ (0.021 s^-1^). The mixed layer deepened and stratification in the upper ocean weakened.
3. There were two weaker cyclonic eddies within the latitude from 12°N to 18°N along the longitude 68°E before the arrival of Ockhi. As the TC passed on December 3, internal gravity waves with an inertial period of approximately 2 days were generated in the thermocline below the mixed layer. After the passage of Ockhi, the two eddies was enhanced.
4. With high PAR and little precipitation, the enhanced cyclonic eddies maintained upwelling for over ten days, enabling a high concentration of nitrates to be uplifted into the mixed layer.

## Supporting information

S1 Data (NC4)

S2 Data (RAR)

## References

[1] Emanuel KA. An air-sea interaction theory for tropical cyclones. Part I: Steady-state maintenance. J Atmos Sci. 1986; 43(6): 585–605

[2] Akter N, Tsuboki K. Role of synoptic-scale forcing in cyclogenesis over the Bay of Bengal. Climate Dynamics. 2014; 43(9–10): 2651–2662.

[3] McPhaden MJ, Foltz GR, Lee T, Murty VSN, Ravichandran M, Vecchi GA, et al Ocean–atmosphere interactions during cyclone Nargis. EOS Trans AGU. 2009; 90(7): 53–54.

[4] Thadathil P, Muraleedharan PM, Rao RR, Somayajulu YK, Reddy GV, Revichandran C. Observed seasonal variability of barrier layer in the Bay of Bengal. Journal of Geophysical Research. 2007; 112: C02009 10.1029/2006JC003651

[5] Girishkumar MS, Ravichandran M, Han W. Observed intraseasonal thermocline variability in the Bay of Bengal. Journal of Geophysical Research. 2013; 118(7): 3336–3349.

[6] Rao RR. Further analysis on the thermal response of the upper Bay of Bengal to the forcing of pre-monsoon cyclonic storm and summer monsoonal onset during MONEX-79. Mausam. 1987; 38:147–156.

[7] Gopalakrishna VV, Murty VSN, Sarma MSS, Sastry JS. Thermal response of upper layers of Bay of Bengal to forcing of a severe cyclonic storm: a case study. Indian Journal of Marine Sciences. 1993; 22(1): 8–11

[8] Subrahmanyam B, Murty VSN, Sharp RJ, O'Brien JJ. Air-sea coupling during the tropical cyclones in the Indian Ocean: a case study using satellite observations. Pure and Applied Geophysics. 2005; 162(8–9): 1643–1672.

[9] Neetu S, Lengaigne M, Vincent EM, Vialard J, Madec G, Samson G, et al Influence of upper-ocean stratification on tropical cyclone-induced surface cooling in the Bay of Bengal. Journal of Geophysical Research. 2012; 117(C12). 10.1029/2012JC008433

[10] Lloyd ID, Vecchi GA. Observational evidence for oceanic controls on hurricane intensity. J Clim. 2011; 24(4):1138–1153.

[11] Yablonsky RM, Ginis I. Impact of a Warm Ocean Eddy's Circulation on Hurricane-Induced Sea Surface Cooling with Implications for Hurricane Intensity. Mon Weather Rev. 2013; 141(3): 997–1021. 10.1175/MWR-D-12-00248.1

[12] Nayak SR, Sarangi RK, Rajawat AS. Application of IRS-P4 OCM data to study the impact of cyclone on coastal environment of Orissa. Curr Sci. 2001; 80(9): 1208–1213.

[13] Vinayachandran PN, Mathew S. Phytoplankton bloom in the Bay of Bengal during the northeast monsoon and its intensification by cyclones. Geophysical Research Letters. 2002; 30(11): 1572 10.1029/2002GL016717

[14] Rao KH, Smitha A, Ali MM. A study on cyclone induced productivity in south-western Bay of Bengal during November-December 2000 using MODIS (SST and chlorophyll-a) and altimeter sea surface height observations. Indian Journal of Geo-Marine Sciences. 2006; 35(2): 153–160.

[15] Madhu NV, Maheswaran PA, Jyothibabu R, Sunil V, Revichandran C, Balasubramanian T, et al Enhanced biological production off Chennai triggered by October 1999 super cyclone (Orissa). Curr Sci. 2002; 82(12): 1472–1479.

[16] Gomes HR, Goes JI, Saino T. Influence of physical processes and freshwater discharge on the seasonality of phytoplankton regime in the Bay of Bengal. Continent Shelf Res. 2000; 20(3): 313–330.

[17] Rao AD, Joshi M, Jain I, Ravichandran M. Response of subsurface waters in the eastern Arabian Sea to tropical cyclones. Estuarine, Coastal and Shelf Science 2010; 89(4): 267–276.

[18] Lin II, Chen CH, Pun IF, Liu T, Wu CCW. Warm ocean anomaly, air sea fluxes, and the rapid intensification of tropical cyclone Nargis (2008). Geophysical Research Letters. 2009; 36(3): L03817 10.1029/2008GL035815

[19] Kaplan J, DeMaria MJ, Knaff A. A revised tropical cyclone rapid intensification index for the Atlantic and eastern North Pacific Basins. Weather Forecast. 2010; 25(1): 220–241.

[20] Wentz FJ, Scott J, Hoffman R, Leidner M, Atlas R, Ardizzone J. Remote Sensing Systems Cross-Calibrated Multi-Platform (CCMP) 6-hourly ocean vector wind analysis product on 0.25 deg grid, Version 2.0. Remote Sensing Systems, Santa Rosa, CA. 2015. Available online at www.remss.com/measurements/ccmp.

[21] Barnier B, Madec G, Penduff T, Molines JM, Treguier AM, Le Sommer J, et al Impact of partial steps and momentum advection schemes in a global ocean circulation model at eddy permitting resolution. Ocean Dynamics. 2006; 59(3): 543–567. 10.1007/s10236-006-0082-1

[22] Benkiran M, Greiner E. Impact of the Incremental Analysis Updates on a Real-Time System of the North Atlantic Ocean. Journal of Atmospheric and Oceanic Technology. 2008; 25(11): 2055–2073.

[23] Chambers DP, Cazenave A, Champollion N, Dieng H, Llovel W, Forsberg R, et al Evaluation of the Global Mean Sea Level Budget between 1993 and 2014. Surveys in Geophysics. 2016; 38(1): 309–327.

[24] Tranchant B, Reffray G, Greiner E, Nugroho D, Koch-Larrouy A, Gaspar P. Evaluation of an operational ocean model configuration at 1/12° spatial resolution for the Indonesian seas–Part 1: Ocean physics, Geosci. Model Dev. 2016; 9(3): 1037–1064.

[25] Wessel P, Smith WHF. A global, self-consistent, hierarchical, high-resolution shoreline database. Journal of Geophysical Research. 2013; 101(B4): 8741–8743. 10.1029/96JB00104

[26] Enriquez AG, Friehe CA. Effects of Wind Stress and Wind Stress Curl Variability on Coastal Upwelling. Journal of Physical Oceanography. 1995; 25(7):1651–1671.

[27] Zhao H, Han G, Zhang S, Wang D. Two phytoplankton blooms near Luzon Strait generated by lingering Typhoon Parma. Journal of Geophysical Research. 2013; 118(2): 412–421.

[28] Chen X, Pan D, He X, Bai Y, Wang D. Upper ocean responses to category 5 typhoon Megi in the western north Pacific. Acta Oceanologica Sinica. 2012; 31(1): 51–58.

[29] Liu YG, Weisberg RH, Zheng LY. Impacts of Hurricane Irma on the Circulation and Transport in Florida Bay and the Charlotte Harbor Estuary. Estuaries and Coasts. 2019; 10.1007/s12237-019-00647-6

[30] He Q, Zhan H, Shuai Y, Cai S, Li QP, Huang G, et al Phytoplankton bloom triggered by an anticyclonic eddy: The combined effect of eddy-Ekman pumping and winter mixing. Journal of Geophysical Research. 2017; 122(6): 4886–4901.

[31] Zhao H, Shao J, Han G, Yang D, Lv J. Influence of Typhoon Matsa on Phytoplankton Chlorophyll-a off East China. PLoS ONE. 2015; 10(9): e0137863 10.1371/journal.pone.0137863 26407324 PMC4583286

[32] Turner J S. Buoyancy effects in fluids 1st ed. New York: Cambridge University Press, 1973.

[33] Geisler JE. Linear theory of the response of a two-layer ocean to a moving hurricane. Geophys. Fluid Dyn. 1970; 1: 249–272.

[34] Wu R, Zhang H, Chen D, Li C, Lin J. Impact of Typhoon Kalmaegi (2014) on the South China Sea: Simulations using a fully coupled atmosphere-ocean-wave model. Ocean Modelling. 2018; 131:132–151. 10.1016/j.ocemod.2018.08.004

[35] Bender MA, Ginis I. Real case simulations of hurricane-ocean interaction using a high-resolution coupled model: effects on hurricane intensity. Monthly Weather Review. 2000; 128(4): 917–946.

[36] Rao AD, Dash S, Babu SV, Jain I. Numerical modeling of cyclone’s impact on the ocean—A case study of the Orissa Supper Cyclone. Journal of Coastal Research. 2007; 23 (5):1245–1250. 10.2112/05-0517.1

[37] Niu L, van Gelder PHAJM, Zhang C, Guan Y, Vrijling JK. Physical control of phytoplankton bloom development in the coastal waters of Jiangsu (China). Ecological Modelling. 2016; 321: 75–83.

[38] Liu YG, Weisberg RH, Law J, Huang BY. Evaluation of Satellite-Derived SST Products in Identifying the Rapid Temperature Drop on the West Florida Shelf Associated With Hurricane Irma. Marine Technology Society Journal. 2018; 52(3): 43–50.

[39] Liu X, Wang M, Shi W. A study of a Hurricane Katrina-induced phytoplankton bloom using satellite observations and model simulations. Journal of Geophysical Research. 2009; 114(C3), 10.1029/2008JC004934

[40] Zheng ZW, Ho CR, Kuo NJ. Importance of pre-existing oceanic conditions to upper ocean response induced by Super Typhoon Hai-Tang. Geophysical Research Letters. 2008; 35(20): L20603, 10.1029/2008GL035524

[41] Zhang SW, Xie LL, Hou YJ, Zhao H, Qi YQ, Yi XF. Tropical storm-induced turbulent mixing and chlorophyll-a enhancement in the continental shelf southeast of Hainan Island. Journal of Marine Systems. 2014; 129: 405–414.

